# Sweet solutions: nectar chemistry and quality

**DOI:** 10.1098/rstb.2021.0163

**Published:** 2022-06-20

**Authors:** Susan W. Nicolson

**Affiliations:** Department of Zoology and Entomology, University of Pretoria, Pretoria 0002, South Africa

**Keywords:** sugar composition, amino acids, secondary metabolites, nectar concentration

## Abstract

Nectar, the main floral reward for pollinators, varies greatly in composition and concentration. The assumption that nectar quality is equivalent to its sugar (energy) concentration is too simple. Diverse non-sugar components, especially amino acids and secondary metabolites, play various roles in nutrition and health of pollinators. Many nectar compounds have indirect effects by altering the foraging behaviour of pollinators or protecting them from disease. This review also emphasizes the water component of nectar, often ignored because of evaporative losses and difficulties in sampling small nectar volumes. Nectar properties vary with environmental factors, pollinator visits and microbial contamination. Pollination mutualisms depend on the ability of insect and vertebrate pollinators to cope with and benefit from the variation and diversity in nectar chemistry.

This article is part of the theme issue ‘Natural processes influencing pollinator health: from chemistry to landscapes’.

## 1. Introduction

Nectar is the most common reward offered by flowering plants to their pollinators. While pollen came first in evolutionary terms, nectar is consumed by a greater diversity of animals, though not all are pollinators. Pollen is released by a flower only once, but nectar is a renewable floral resource, being secreted daily in long-lived flowers or replenished after removal [1]. Nectar is a complex and dynamic fluid [2–5], described as ‘notoriously plastic’ [6]. Nectar quantity and quality (volume and chemical composition) may vary greatly from the initial offering as the flower ages, when visitors remove it or add microbes, or as the weather changes. This review highlights the complexity of nectar chemistry and its contribution to the nutrition and health of insect and vertebrate pollinators. The focus will be on floral nectar, but extrafloral nectar has many similarities [4].

The chemical complexity of nectar was recognized in the 1970s in the pioneering work of Herbert and Irene Baker, who drew attention to the variety and ubiquity of non-sugar solutes (for review see [7]). Measured values for chemical composition were generally assumed constant for a species, which encouraged the search for patterns. The relative influences of phylogeny and pollinator type on sugar composition became the subject of many studies, often based on single or a few nectar samples for each species, or pooled samples if the volumes were insufficient for analysis. Pooling obscures variability in nectar sugars at the species level, usefully highlighted in *Helleborus foetidus* [8]. Phenotypic variation in nectar traits is common both within and between populations [6]. For example, variation in sugars and amino acids is particularly high among flowers on individual plants of the orchid *Gymnadenia conopsea* [9]. In this study, 45% of the variance in nectar traits was found among flowers within individuals and 20% between populations. Sampling of field populations of *Polemonium caeruleum* contradicted an earlier report, based on a single population, of high nectar sucrose and proline levels [10]. Nectar solutes have been compared in multiple species or cultivars grown from seed either outdoors or in greenhouses [11,12]. The simplified greenhouse environment reduces variation in nectar chemistry resulting from biotic and abiotic factors [13] but is less ecologically relevant. Recent studies using transcriptomics and untargeted metabolomics to elucidate the mechanisms of nectar secretion are leading to the discovery of previously unreported nectar metabolites [14,15].

Nectar quality is usually defined by its sugar concentration, for pollinators as diverse as bees and bats [16,17]. Nectar quantity refers to its volume, which tends to be inversely proportional to concentration (but see [18] on hummingbird flowers). Nectar volumes can range from less than a microlitre in many bee flowers to several millilitres in flowers pollinated by birds or bats, and sugar concentrations in nectar range from about 10–70% w/w. These easily measured nectar traits can be combined to calculate the sugar content per flower, a better measure of the energetic value [19]. Sugar production per 24 h can be scaled up to landscape level by multiplying by flower density [20–22]. In practice, there are many complications in sampling nectar as described above [6,19,23]. Moreover, the presence of non-sugar components leads to overestimates in ‘sugar’ concentrations when refractometers are used [24,25]. Nectar is too complex and variable for a narrow definition of quality with a strictly energetic focus: the energy from sugar, while crucial [26], is not the only benefit to pollinators (table 1).

**Table 1.** Consequences of nectar chemistry for pollinator health.

nectar components	effects on pollinator health	references
sugars	energy source for flight, thermoregulation and development, including endotherms with high metabolic rates	[26]
	some birds (e.g. starlings) cannot digest sucrose	[27]
	glucose and fructose reduce oxidative damage in hawkmoths	[28]
amino acids	non-essential amino acids often predominate and ratios vary greatly; nutritional benefit is largely unknown	[29]
	may affect the taste of nectar	[30]
	amino acid preferences may influence sugar intake	[31,32]
	metabolized in flight	[33,34]
	pharmacological effects of non-protein amino acids may benefit plant but not pollinator	[4,35]
proteins	nectar preservatives	[36]
fatty acids	metabolised by hawkmoths	[34]
salts	contribute to salt balance	[24]
	high K+ is deterrent (e.g. onion); may affect energy intake	[37,38]
vitamin C (ascorbic acid)	reduces oxidative damage	[39]
secondary metabolites	nectar preservatives	[40]
	antioxidants, e.g. phenolics	[41]
	antiparasitic action on *Crithidia* in bumblebees or *Nosema* in honeybees	[42–45]
	pharmacological effects of caffeine and nicotine lead bees to overvalue nectar quality	[35,46,47]
	deterrents to nectar robbers or competing pollinators; preferred pollinator access is enhanced	[48,49]
	quercetin upregulates detoxification genes	[50]
water	excess water in dilute nectars must be removed	[51,52]
	viscosity affects drinking rates	[53]
	water source in dry environments	[23]

## 2. Sugars

Most of the nutritional value of nectar is owing to three simple sugars—sucrose and its component hexoses, glucose and fructose. Nectar sugars originate from sucrose uploaded from phloem to nectary tissue. Sucrose from photosynthesis may be temporarily stored as starch in the nectary and later degraded: this enables faster nectar secretion, as in the large volumes of nectar produced by *Cucurbita pepo* (squash) flowers [14,54]. Analyses of phloem sap and nectar of the same plant show that sucrose is broken down to varying extents by cell-wall invertases during nectar secretion [11,55,56]. This regulates the relative amounts of the three sugars [2,14,57] and maintains the sucrose concentration gradient [57]. Water influx owing to the higher osmolality of hexose solutions will lead to copious and extremely dilute nectars, such as those of *Aloe* and *Erythrina* species adapted to generalized bird pollinators [58,59]. A recent model of nectar secretion shows how modulation of cell wall invertase activity accounts for differences in both nectar volume and sugar composition [60]. Hydrolysis of sucrose should result in a 1 : 1 ratio of glucose and fructose, but an imbalance in fresh nectar indicates the role of other biochemical pathways. The sugar profile may also be modified by nectar microbes (see below). Reabsorption of sugars may contribute to homeostasis or recover the investment in nectar, but the process is poorly understood [2,54,61].

Other sugars in nectar occur in negligible amounts compared to the three dominant sugars [7,14,39,62]. For example, maltose comprised 2.5% of nectar sugars in *P. caeruleum* but was absent in some populations [10]. However, the pentose sugar xylose represents up to 39% of total sugars in *Protea* and *Faurea*, two sister genera of the Proteaceae [63]. Xylose is metabolized by mammal pollinators [64] and is prominent in the dilute nectars of beetle-pollinated *Protea* species [65].

The relative proportions of sucrose, glucose and fructose in nectars of different plant species have often been thought to be correlated with pollination syndromes [66]. However, there is frequently a strong association with plant phylogeny. Studies that have examined associations of nectar sugar composition with pollinator groups and plant phylogeny, usually with a geographical focus, are reviewed in [39]; and the historical debate continues. For bird-pollinated flowers, nectar properties of more than 500 species in Africa and the Americas have been examined, and phylogenetic analysis showed that the dichotomy in nectar sugars (sucrose or hexose) is not between plants pollinated by hummingbirds and passerine birds but rather between specialized and generalized nectarivores [59]. This division into specialist and generalist pollinators is supported by a comprehensive study [67] that examined more than 2000 species of asterids (this clade accounts for a quarter of angiosperm species diversity). Despite much variation, the data showed evolution towards two optimal values for nectar sucrose proportion: low sucrose in nectars consumed by generalist birds and unspecialized insects, high sucrose in other pollinator groups. The authors concluded that the hypothesis of adaptation to pollinator group is only a partial explanation for sugar composition. However, another analysis of nectar sugars extending across the angiosperms (over 1200 species) and using a different modelling approach found low phylogenetic signal and confirmed the importance of pollinator type [68].

It can be argued that the perceived association with pollinator type is a secondary consequence of flower morphology [23]. Sucrose-rich nectars are more common in protected, tubular flowers visited by specialist pollinators (e.g. the heather family Ericaceae), while hexose-rich nectars dominate in open exposed flowers visited by both generalists and specialists (e.g. the daisy family Asteraceae) [67]. This correlation, recognized long ago [69] in a semi-quantitative study of nectar sugars in 900 plant species, can be explained by physical chemistry. Hexose nectars have much higher osmotic concentrations than the equivalent sucrose nectars; therefore they evaporate more slowly and are in better balance with dry air than sucrose nectars, which need protection in long corollas [70,71]. Long-tongued pollinators which can access the nectar in protected flowers, such as bees, moths and butterflies, will then appear to prefer sucrose nectars.

Nectar sugars in bird-pollinated flowers are linked to feeding choices and digestive constraints. If nectar sucrose is not hydrolyzed in the flower, it must be hydrolyzed in the gut of the pollinator before absorption. The invertases in plant nectaries are β-fructosidases, while animals use *α*-glucosidases, present in honeybees and nectar-feeding birds [27,41]. Intestinal sucrase activity in nectar-feeding birds matches the proportion of sucrose in the nectars they consume: this convergent coevolution is seen across continents in hummingbirds, sunbirds and honeyeaters, and the flowers they pollinate [27]. In spite of this association, these birds do not prefer sucrose to hexose nectars, and even prefer hexose nectars at low concentrations [72]. The methods used in preference tests are important because of the caloric and osmotic differences between sucrose and hexose nectars: hexose solutions mixed on a percentage weight basis have 95% the energy value of sucrose solutions [72,73]. Ultimately, the sugar composition of nectar may not be physiologically important, because pollinators digest sucrose rapidly, and nectar sugars are efficiently assimilated [74,75]. The few exceptions include starlings and their allies and some acacia ants, which lack sucrase and avoid sucrose-containing floral and extrafloral nectars respectively [27,76]. Hummingbirds and bats use both fructose and glucose directly to support their high metabolic rates in hovering flight [77]. In hawkmoths, nectar glucose protects against the oxidative stress of flight [28].

Nectar is a sugar-rich medium for microbial growth, and colonization by yeasts and bacteria changes nectar chemistry. Nectar microbes are dispersed mainly by floral visitors, especially in long-lived flowers with a lot of pollinator traffic. Nectar robbers may contribute to even higher levels of microbial abundance [78]. Sugars are the primary energy source for microbes as well as pollinators. The result is a lower nectar sugar concentration and a smaller proportion of sucrose, in direct proportion to the density of yeast cells [79]. Selective consumption of glucose may cause fructose to dominate the nectar sugars. Microbes also use amino acids as a nitrogen source in nectar and change its pH [80,81]. In addition to reducing its nutritional value, microbial metabolism may add new compounds to nectar [82]. An extreme example is the high ethanol content in fermented yeast-containing nectar consumed by treeshrews in Malaysian rainforests [83].

## 3. Amino acids

Amino acids occur in nectar at far lower concentrations than sugars. Total amino acids are in the micromolar to millimolar range; for comparison, a 30% w/w sucrose solution is 1 molar. Total amino acids in the nectar of 30 insect-pollinated species in the UK ranged from 0.19 to 12.7 mM [84]. Parallel analyses of nectar and phloem sap of oilseed rape (*Brassica napus*) and other species have shown that, while total sugar concentrations in nectar and phloem sap are similar, the total amino acid concentration in nectar is two orders of magnitude lower than in phloem sap [11,55]. Amino acids are thus retained in the nectary during nectar secretion.

More important than the total concentration is the amino acid composition, which varies greatly in nectars of different plant species [84,85]. All 20 amino acids commonly found in proteins are present in nectar. Sometimes the amino acid profile is heavily skewed towards non-essential amino acids, which predominate in phloem sap. Four amino acids—glutamine, glutamate, asparagine, aspartate—are important in the nitrogen metabolism of plants (some have high N : C ratios) and are relatively abundant in nectar, together with alanine, serine, glycine and proline, also non-essential amino acids [12,86]. Transcriptomics of the cotton nectary showed high expression of genes that use glutamate as a substrate for the biosynthesis of other amino acids such as aspartate [15]. On the other hand, one or two essential amino acids can dominate the amino acid profile. Phenylalanine is abundant in the nectars of mainly bee-pollinated Mediterranean plants, representing 47% of total amino acids in Lamiaceae [85]. Essential amino acids as a percentage of total amino acids vary widely, for example from 6–48% in 20 *Nicotiana* species [12]. Non-protein amino acids, such as taurine, *γ*-aminobutyric acid (GABA) and *β*-alanine, occur at high concentrations in certain nectars and are sometimes classed as secondary metabolites [87].

Pollen is a potential source of nectar amino acids, especially as it is far richer in these compounds, e.g. 1000× higher than in nectar on a per flower basis [88]. Contamination with pollen falling into nectar could lead to high levels of proline and other amino acids [89,90]. While deliberate exposure to pollen did not increase amino acids in nectar of *Aloe marlothii* [86], sunflower pollen added to synthetic nectar leached amino acids, and pollen contamination in visited flowers of *Gentiana lutea* enriched the amino acid profile in nectar [90,91]. Recently, bacteria in nectar have been shown to induce pollen germination and bursting, thus increasing protein levels [92]. The risk of contamination is high for flowers with low nectar volumes when sampling method is critical for amino acid analyses [93].

The functional significance of nectar amino acids for the health of pollinators is not clear. Their nutritional value is probably small for most pollinator groups. Baker & Baker [94] looked for associations between pollinator type and nectar amino acids, suggesting that flowers pollinated by Lepidoptera had higher nectar amino acids because adults lack other nitrogen sources [29]. However, no relation between amino acid concentration or composition and pollination syndrome is evident in the diverse genus *Impatiens*, including butterfly pollinated species [95]. Bees and hoverflies obtain amino acids from pollen, while nectar-feeding birds use arthropods and sometimes pollen as protein sources. It is not clear why *Erythrina* species pollinated by passerine birds have much higher amino acid concentrations than hummingbird species [86,96]; sunbirds (*Cinnyris talatala*) do not prefer artificial nectar containing amino acids [97].

Apart from direct nutritional benefits, amino acids may contribute to the taste and attractiveness of nectar [30], and to the foraging choices of pollinators. This is complicated by the large number of compounds: individual amino acids may have attractive or repellent effects that are obscured in a mixture [98]. Interestingly, a newly identified taste receptor in the honeybee responds to glutamate, aspartate, asparagine and glutamine, the main amino acids of nitrogen transport in plants and relatively abundant in nectar [99]. The many studies on the neural and behavioural responses of pollinators (mainly bees) to specific amino acids or to amino acid mixtures are beyond the scope of this review. Non-protein amino acids can be surprisingly abundant in nectar and also modulate insect behaviour by acting as neurotransmitters, as do glutamate and glycine [35,87]. The case of proline is interesting: it is common in nectar, the most abundant amino acid in the haemolymph of honeybees and used in early phase flight of bumblebees and wasps [33,100]. Hawkmoths also use amino acids as metabolic fuel [34].

The presence of amino acids may affect sugar preferences and intake of pollinators. Free-flying honeybees select lower sucrose concentrations when attracted by phenylalanine (known to be phagostimulatory), or higher concentrations to offset the deterrent effect of glycine [31]. Sugar concentration preferences of the hawkmoth *Manduca sexta* are modified by the presence of an amino acid blend resembling that in natural nectar [32]. Similarly, nectar amino acids reduce discrimination between sugar concentrations in bats [101]. From the plant perspective, this may conserve nectar sugars.

## 4. Micronutrients and minor metabolites

Beyond targeted analyses of the two main classes of metabolites, sugars and amino acids, nectar chemistry is less clear. Lipids, organic acids, minerals and proteins occur in nectar at low concentrations, but quantitative information is mostly fragmentary [39]. As in the case of amino acids, little is known of the origin of these non-sugar metabolites in nectar. Recently, the untargeted metabolomics approach has revealed a great diversity of metabolites, for example in floral and extrafloral nectar of cotton and floral nectar of squash [14,15].

The inorganic ion content of nectar is generally overlooked, compared to that of pollen. As with amino acids, it is assumed that pollinators supplement their mineral intake by consuming arthropods or pollen. However, minerals in some nectars may contribute to salt balance [24,39]. Comprehensive analyses of the nectar chemistry of 20 *Nicotiana* species and 147 species of Bromeliaceae showed that the average total millimolar concentration of inorganic ions was higher than that of amino acids [12,102]. Unusually high potassium concentrations in onion and avocado nectar are repellent to honeybees, leading to poor pollination [37,103]. High potassium and phosphate levels in feeder solutions deterred honeybees but not native pollinators of avocado in Mexico [38], and their deterrent effect has been confirmed using proboscis extension responses of honeybees engaged in water foraging [104]. Salt regulation in honeybees is discussed in [105].

Other nectar constituents are proteins, lipids and organic acids. Nectar proteins (nectarins) protect floral and extrafloral nectar from microbial degradation [40]. In the copious nectar of ornamental tobacco nectarins contribute to generating hydrogen peroxide at levels up to 4 mM through the nectar redox cycle [36]. One of the nectar proteins in *Jacaranda mimosifolia* is a lipase that hydrolyses nectar lipids to free fatty acids, which accumulate to 0.6 mM and may be attractive to bees [106]. Nectar fatty acids may have a metabolic role; hawkmoths used palmitic acid in artificial nectar as fuel for resting metabolism [34]. In *Nicotiana* species, only one organic acid, malic acid, was present at significant concentrations (up to 2 mM) [12]. Ascorbic acid (vitamin C) plays a role in the redox cycle and is well known as an antioxidant in nectar [39].

## 5. Secondary metabolites

Secondary metabolites, such as alkaloids, flavonoids, terpenoids and phenolics, are common in the nectar of plants that produce them as defence against herbivore attack [42,107]. These nectar components may influence foraging behaviour and override nutritional benefits. In any plant species, the compounds present in nectar, pollen and other plant tissues are chemically similar, although the actual compounds may differ and pollen has more chemical diversity and higher concentrations of defensive chemicals than nectar [43,108]. The systematic look at chemistry of floral rewards and its variation in 31 plant species carried out by Palmer-Young *et al*. [108] represents the first non-targeted metabolomics approach to analysing secondary metabolites in nectar and pollen. While it seems paradoxical that nectar contains deterrent or toxic compounds, the concentrations can be highly variable and the ecological consequences are dependent on the dose (it is important to test multiple concentrations) and on the context (for example, availability of other forage sources). Subtle beneficial effects may become apparent at lower concentrations, with varying consequences for plant-pollinator interactions (reviewed by [42]). Manipulation of pollinator behaviour may improve the reproductive success of plants, and protection against disease may help the fitness of pollinators.

Because sensitivity to secondary metabolites varies among floral visitors, unpalatable compounds may function as taste filters that screen out ineffective pollinators and nectar robbers. Two South African examples are the dark phenolic-containing nectar of *Aloe vryheidensis*, which deters honeybees and sunbirds, but not generalist bulbuls, and the unpalatable nectar of a milkweed that is preferentially consumed by spider wasps [48,109]. Grayanotoxins in nectar of the invasive *Rhododendron ponticum* in the UK are toxic to honeybees but not to the main bumblebee pollinator *Bombus terrestris* [49]; geographical variation in the filtering function of these compounds has consequences for invasion biology [110]. Deterrence depends on concentrations of both sugar and toxin: honeybees and nectar-feeding birds are more tolerant to nicotine in artificial nectars of higher concentration [111,112].

Even low concentrations of secondary metabolites can influence pollinator behaviour. Caffeine is present in nectar of *Coffea* and *Citrus*, which have flowers highly attractive to bees [46]. Honeybees fed caffeine at concentrations that were ecologically relevant but below their taste thresholds had improved memory for the associated floral scent. Caffeine thus caused them to overestimate the quality of the nectar. A field study confirmed this, with dramatic increases in colony-level recruitment to caffeine-laced sucrose solutions, leading to suboptimal foraging strategies [113]. Similarly, bumblebees tolerate low calories for nicotine [47]. These pharmacological manipulations of pollinator behaviour may benefit the plant through enhanced pollen transfer but are unlikely to benefit pollinators. In a very different example of pollinator attraction through secondary metabolites, coloured nectar tends to be associated with vertebrate pollinators, often on islands [114], and it has now been shown that the blood-red nectar of flowers attractive to geckos is owing to an alkaloid pigment, nesocodin [115].

Nectar secondary metabolites can protect pollinators against parasites and pathogens. Health-promoting effects of consuming secondary metabolites have been demonstrated in several studies on *Bombus* species and their gut parasites (reviewed in [42]). Generally the protective mechanism is not understood, but an exception is callunene in nectar of the heather *Calluna vulgaris* [44]. This compound, identified when honey extracts from important bee plants were screened for their activity against the intestinal parasite *Crithidia bombi*, removes the flagellum anchoring the parasite to the bumblebee hindgut. In honeybees, dietary caffeine reduces spore loads of the protozoan *Nosema ceranae* but nicotine does not [45,116]. Whether secondary metabolites in nectar can clear or prevent infections may depend on modification by the gut microbiome [117]. Other health benefits include the well-known antioxidant effects of phenolics in honey, which depend on the nectar source [41]. The flavonoid quercetin is common in nectar as well as in pollen, preferred by honeybees in preference tests, and upregulates detoxification genes [50,108,118]. For most pollinators, the health benefits of secondary metabolites are unknown.

Microbial communities in nectar may be limited by secondary metabolites. Potential antimicrobial effects were examined in almond, citrus and tobacco nectar [119]; while bacterial communities differed between these nectars, their growth was only weakly inhibited by the respective secondary metabolites amygdalin, caffeine and nicotine. Conversely, microbes can reduce the levels of some secondary metabolites in nectar [120]. The relatively constant sugar composition of some nectars suggests a role of secondary metabolites as nectar preservatives, but more research is needed, including consideration of possible synergistic effects [40].

## 6. Water

Water is a nutrient, unlike the nectar solutes discussed above—proteins, non-protein amino acids and secondary metabolites—which has no direct function in nutrition. The water component of nectar is seldom emphasized in the nectar chemistry literature. This is partly because of the variability in water content associated with environmental conditions. Also, small volumes of many insect-pollinated flowers require that nectar is collected by the wick method or by rinsing, which provides no information on volume or water content.

Nectar concentration is greatly influenced by floral microclimate. Unless protected, nectar tends to equilibrate with ambient humidity and this results in evaporation in all but very humid conditions. It is seldom appreciated that a 20% sucrose solution will lose water to air at all humidities below 98% [70]. The speed of evaporation depends on floral morphology, the microclimatic gradients in and around flowers and the sugar profile, being slower from hexose nectars. It is also faster when the nectar volume is small because a small droplet has a larger surface area. Evaporation from open flowers, along with discontinuous secretion, possible reabsorption and periodic removal by floral visitors, may lead to great diurnal variation in nectar volumes and concentrations in a single species, and in the attractiveness to different pollinators. However, nectar in exposed flowers is often more dilute and abundant than predicted and may be a water source in dry environments [23,121].

Nectar consumed by pollinators is often more dilute than the synthetic nectars they choose in preference tests. Bats provide an interesting example. The classic bat pollination syndrome includes abundant and highly dilute hexose-rich nectar, averaging 17% w/w [122]. In choice tests, bats prefer much higher concentrations: a suggested explanation for the discrepancy, based on experiments with free-flying bats in Costa Rica, is that competition for food causes bats to look for higher volumes and to be less discerning about concentration [17]. This work is, however, controversial [123]. Bumblebees on artificial flowers respond more readily to increased concentration than to volume: in this situation (and perhaps in real flowers) concentration may be a more reliable and easily assessed cue [124]. Pyke *et al.* [125] argue that evolution should result in nectar concentrations that benefit individual plants rather than pollinators, and that plants use a combination of nectar attributes (volume, concentration and composition) to manipulate pollinators.

Dilute nectars offer an important viscosity advantage. The viscosity of sucrose solutions increases exponentially with concentration and has a marked effect on the ease of drinking nectar. Optimal nectar concentrations for different pollinator groups depend on their drinking technique, with suction feeders requiring lower concentrations than lapping feeders [53]. Honeybee tongues function as hairy mops, and the lapping frequency remains constant on solutions of equal viscosity but differing concentration [126]. Surprisingly, individual bees are able to switch feeding modes from lapping to suction at nectar concentrations below 30% w/w, enabling faster energy intake [127]. The behavioural adaptability of bees in dealing with different nectar concentrations is also evident when they remove excess water through repeated regurgitation and evaporation. This may begin during foraging in honeybees, which arrive back at the hive with crop sugar concentrations that are double those in nectar [51]; useful in view of the considerable cost of processing nectar into honey [128]. Both social and solitary bees concentrate nectar on their tongues [129]. For nectar-feeding birds, processing excess water is a physiological challenge. They excrete the excess (hummingbirds) or simply do not absorb it (sunbirds and honeyeaters) (reviewed by [52]).

## 7. Concluding remarks

There are several difficulties with using sugar concentration as a measure of nectar quality. Non-sugar ingredients are chemically complex and diverse, with functions that are not only nutritional (table 1). Nectar compounds are usually studied in isolation, and interactive effects on pollinator responses, e.g. phenolics and potassium in onion nectar [130], have scarcely been considered. Nectar exhibits great variability owing to genetic and environmental factors, removal by pollinators and contamination with pollen and microbes during pollinator visits. Apart from getting enough calories in nectar, the health of pollinators depends on their ability to deal with varying chemical composition and fluctuating concentrations. The value of nectar depends equally on its quantity (volume per flower and flower density), and the availability of nectar ultimately overrides quality considerations.

The need for diversity in pollinator diets (diet breadth) is frequently stressed in the context of bee population declines, with the emphasis usually on pollen. Mass-flowering monocultures in agricultural landscapes lack diversity and complementary food sources are necessary, but there is scope for improvement in the quality of these short-lived nectar resources. Plant breeding has led to much variation in nectar-related traits, although pollination is not the focus of breeding programmes [131]. Nectar and pollen of different genotypes of field bean *Vicia faba* vary greatly and, in view of bee preferences, breeding for higher nectar concentrations may be more desirable than for higher nectar volumes [132]. In another bee-pollinated crop, oilseed rape, field-grown cultivars showed striking variability in nectar volumes, compared to sugar or amino acid composition [11]. In cultivated sunflowers, floret size is critical for easy access of bees to nectar [131,133]. Secondary metabolites may also change with domestication, and nectar of blueberry cultivars has reduced levels of a caffeic acid ester: these compounds protect bumblebees from infection by pathogens [134]. Secondary metabolites in nectar of pollinator-dependant crop plants vary more across cultivars than in pollen, and apple (*Malus domestica*), for example, shows extreme chemical separation across cultivars [108]. Selection on nectar-related traits has the potential to both benefit pollinators and enhance crop pollination [6,131].

Future research on nectar chemistry must include the effects of anthropogenic climate change, for both wild plants and pollinator-dependent crops. Space permits mention of just two studies involving the effects of multiple abiotic factors on nectar properties. Warming, atmospheric CO_2_ and nitrogen enrichment have complex interactive (and sometimes antagonistic) effects on nectar sugars and amino acids of pumpkin *Cucurbita maxima* [135]. Temperature rise and water shortage have varied effects on floral resources of *Borago officinalis*: both stresses decrease nectar volume and thus total sugars, both increase the total nectar amino acids and change their composition, but pollen is more affected by high temperature than by drought [88]. For a review of the metabolic changes occurring in flowers in response to climate change, see [136]. Finally, while elevated atmospheric CO_2_ may dilute protein levels in pollen [137], the relative excess of soluble carbohydrate could make nectar sugars cheap to produce. There is much to learn about the effects of abiotic stresses on nectar production and composition.

## Data Availability

This article has no additional data.
